# Discrimination between two different grades of human glioma based on blood vessel infrared spectral imaging

**DOI:** 10.1007/s00216-015-8891-z

**Published:** 2015-07-14

**Authors:** Katia Wehbe, Isabelle Forfar, Sandrine Eimer, Gianfelice Cinque

**Affiliations:** Diamond Light Source, Harwell Science and Innovation Campus, Didcot, Oxfordshire OX11 0DE UK; INSERM U1034, Université Bordeaux, 33600 Pessac, France; Department of Pathology, Pellegrin University Hospital, 33076 Bordeaux, France

**Keywords:** Glioma, Grading, Blood vessels, FTIR spectroscopy, FPA imaging

## Abstract

Gliomas are brain tumours classified into four grades with increasing malignancy from I to IV. The development and the progression of malignant glioma largely depend on the tumour vascularization. Due to their tissue heterogeneity, glioma cases can be difficult to classify into a specific grade using the *gold standard* of histological observation, hence the need to base classification on a quantitative and reliable analytical method for accurately grading the disease. Previous works focused specifically on vascularization study by Fourier transform infrared (FTIR) spectroscopy, proving this method to be a way forward to detect biochemical changes in the tumour tissue not detectable by visual techniques. In this project, we employed FTIR imaging using a focal plane array (FPA) detector and globar source to analyse large areas of glioma tumour tissue sections via molecular fingerprinting in view of helping to define markers of the tumour grade. Unsupervised multivariate analysis (hierarchical cluster analysis and principal component analysis) of blood vessel spectral data, retrieved from the FPA images, revealed the fine structure of the borderline between two areas identified by a pathologist as grades III and IV. Spectroscopic indicators are found capable of discriminating different areas in the tumour tissue and are proposed as biomolecular markers for potential future use of grading gliomas.

**Electronic supplementary material:**

The online version of this article (doi:10.1007/s00216-015-8891-z) contains supplementary material, which is available to authorized users.

**Graphical Abstract:**
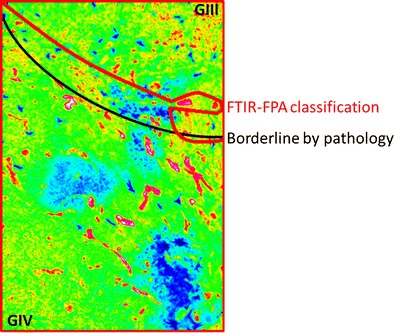
Infrared imaging of glioma blood vessels provides a means to revise the pathologists’ line of demarcation separating grade III (GIII) from grade IV (GIV) parts.

## Introduction

The most common central nervous system tumours in adults are malignant gliomas. Considered the deadliest of human cancers, they are characterized by a highly proliferative index, aggressiveness and invasiveness, leading to short patient survival [1]. Gliomas are classified into four grades with increasing malignancy from I to IV. At grade IV, glioblastomas are among the most vascularized human tumours and may develop de novo as primary glioblastoma or through progression from the lower grade [2, 3]. The fact that microvascular proliferation cannot be observed in low-grade gliomas has led to the hypothesis that both the development and the progression of malignant gliomas largely depend on angiogenesis, i.e. representing a hallmark of the disease [4–6]. Current imaging techniques based on visible contrast microscopy with chemical staining do not always allow for differentiating gliomas from non-tumoural lesions or high-grade tumours from lower-grade lesions, and this can severely impact the patient care. Following the tumour resection, extemporaneous tissue samples are analyzed by the anatomo-pathologist who will need to make a diagnosis of the tumour criteria, in order to give rapid information to the neurosurgeon during the operation that will help the patient for getting the best treatment. Currently, the *gold standard* for prognosis remains to be the expert opinion of pathologists, who visually examine samples of the histological tissue sections deposited on microscope slides. Typical histological classification is based on key criteria, such as cellular density, nuclear atypia, mitotic activity, necrosis and microvascular proliferation [7]. In spite of using stained sections, sometimes it is difficult for the pathologist to strictly define the grade of a tumour; as a consequence, around 30 % of samples are pathologically misclassified due to their heterogeneity [8]. In this sense, we believe that a complementary approach based on rapid and quantitative analysis could provide clinicians with a helpful and powerful tool to more accurately classify tumourous areas, for a quicker diagnosis and easier grading of such an aggressive disease.

Fourier transform infrared (FTIR) microspectroscopy is a well-known technique that allows for qualitative imaging and quantitative analysis of the basic molecular components of biological tissues. Moreover, this technique is quickly establishing its efficacy as a tool for molecular histopathology by detecting subtle chemical changes in human tissue samples [9, 10]. It has been demonstrated that FTIR imaging of tumour tissue combined with advanced chemometrics for spectral data processing can highlight significant spectral changes related to molecular inter- and intra-tumoural heterogeneity not detectable by conventional haematoxylin-eosin (H&E) or immunohistochemistry (IHC) staining [11, 12]. In previous studies, we focused our work on differentiating normal and tumour vasculature of human gliomas by FTIR imaging [13]. In the same way, the analysis of subtle modifications of biomolecule content in the blood vessels (BVs) could reveal tumour growth in different stages [14]. We also studied BV microstructure using conventional versus synchrotron source and combined FTIR microanalysis with chemometrics to well define the vessel structure [15]. In the present work, we used FTIR imaging with a focal plane array (FPA) detector and conventional source to study BVs in tumour tissue sections. FPA technology allows rapid collection of thousands of IR spectra on large tissue sample areas [16–18], and this makes it a potential tool for clinical diagnosis and medical assessment, especially in these kinds of tumour where the rapid answer is essential for the neurosurgery outcome. First, we assessed the FPA performances using different binning modes by quickly screening mouse tumour tissue sections as a test to determine which binning option is the best. Then, we were particularly interested in acquiring FPA images of human glioma sections from areas described as grade III (GIII) and grade IV (GIV) in the same sample. Tumour areas of the two different grades were assessed by pathologists on parallel sections stained with H&E. The aim is to establish features of the vascularity biomolecular markers of the two grades. The most interesting finding in this work is that FTIR-FPA imaging could provide a means to revise the pathologist’s line of demarcation separating GIII from GIV parts of a single section.

## Materials and methods

### Samples

Mouse xenografted tumours (resulting from subcutaneous transplantation of rat C6 glioma cells) 3 weeks post-implantation were cryo-microtomed (see [14] for full experimental details). Sections of 10 and 20 μm in thickness were transferred, respectively, to microscope slides for IHC staining and to a zinc selenide (ZnSe) support for FTIR spectral imaging. Human extemporaneous sample was obtained from a patient with glioblastoma. The patient consent was fully obtained. Twenty-micrometre-thick cryo-fixed tissue sections were prepared by the Pathology Department of Bordeaux University Hospital and deposited on silicon (Si) wafers for IR measurements. Serial sections of 10 μm in thickness were deposited on microscopy slides for H&E and IHC staining. Tissue sections were dried in air before FTIR microanalysis. For revealing blood vessel location in parallel tissue sections and defining the regions of interest, IHC using CD31 antibody (for mouse tumour section) or CD34 antibody (for human tumour section) was performed (as previously described in [13] and [14]). All experiments performed in this work were achieved in compliance with the relevant bioethics laws and institutional guidelines in France.

### FPA detector and binning

FPAs are multi-element mercury cadmium telluride (MCT) detector arrays consisting of *n* × *n* pixels (where *n* = 16, 32, 64, 128 and 256 as per current commercial technology). In contrast to a single-element MCT detector, FPAs acquire simultaneously as many spatially resolved spectra as the detector elements. The result of one single measurement is a *spectra matrix* analogous to the detector element matrix. The FPA detector used in this study consists of 64 × 64 elements, for which the pixel physical size is 40 × 40 μm^2^. The final measured area or field of view (FoV) depends on the actual FPA size and magnification optics. In our case, the overall detector area is 2560 × 2560 μm^2^, which by the ×15 Cassegrain objective and matching condenser, used for sample collection in transmission mode, provides a FoV of circa 170 × 170 μm^2^ per one FPA tile. The pixel size on the sample is scaled by the optics too; by the ×15 Cassegrain objective/condenser, the effective pixel size over the sample (i.e. optical sampling) is 2.6 μm. The binning function is a pre or post process intended for IR maps measured with an FPA detector. It calculates the average spectrum of *n* × *n* spectra in a matrix and replaces them with the calculated average spectrum. In this way, the data quality can be significantly improved by reducing the spectral noise but at the cost of spatial resolution; i.e. with 2 × 2 pixel binning, the effective pixel size at the sample become 5.3 μm instead of 2.6 μm with the ×15 optics and the FPA used here. Another effect of the binning function is a smaller file size since the number of spectra acquired is reduced. If each group of e.g. 2 × 2 or 4 × 4 spectra of the spectra matrix is replaced with its average spectrum, the S/N ratio improves by factor 2 or 4 and so on. Despite this positive effect, one should take into consideration that the spatial resolution will be correspondently reduced by factor 2, 4, etc., at the same time.

### FTIR measurements

Images of samples were acquired using a Bruker system (Bruker Optics, UK) available at the MIRIAM beamline B22 at Diamond Light Source. The system consists of a Vertex 80v spectrometer coupled to a Hyperion 3000 microscope attached to the FPA detector described in the previous paragraph. Spectra were collected in transmission mode with the ×15 optics at 4 cm^−1^ as spectral resolution and 32 scan co-additions for the human tissue section (vs 128 background scans) and 8 sample scans for the mouse tissue section (vs 8 background scans). Mouse tumour section was imaged by 80 FPA tiles (*X* = 16 and *Y* = 5). Different areas of the human sections were imaged, namely two small areas by 35 FPA tiles (*X* = 5, *Y* = 7) and 42 FPA tiles (*X* = 6, *Y* = 7) and a bigger area by 228 FPA tiles (*X* = 12, *Y* = 19). IR maps were acquired with and without pixel binning. Images from the mouse tumour section served as a model for testing pixel binning to assess spectral quality versus good spatial resolution. For this sample, we tried different binning modes (2 × 2, 4 × 4 and 8 × 8). Small images from the human section were acquired without pixel binning. However, since the bigger image was a large area of circa 6 mm^2^, it has been binned 2 × 2 at acquisition to keep a high spatial resolution without exceeding the detector holding time for cooling (about 5 h). Table 1 summarizes the binning mode including the pixel size and the acquisition time for each image.

**Table 1:** Summary of the acquired images by FPA

Images	FPA tiles (*X* × *Y*)	Binning mode	Pixel size at sample (μm)	Acquisition time
Mouse image 0	80 (5 × 16)	No binning	2.6	1 h and 10 min
Mouse image 1		2 × 2	5.3	30 min
Mouse image 2		4 × 4	10.6	20 min
Mouse image 3		8 × 8	21.3	19 min
Human image S1	42 (6 × 7)	No binning	2.6	1 h and 10 min
Human image S2	35 (5 × 7)	No binning	2.6	1 h
Human bigger image	228 (12 × 19)	2 × 2	5.3	3 h

### FTIR spectral data analysis

Spectra of BVs were retrieved from integration false colour maps based on the ratio of protein (1700–1500 cm^−1^) to the CH stretching region (3000–2800 cm^−1^) as this helped to reveal BV structure (since this is not always evident in the unstained tissue section). The maps were correlated with the images from IHC/H&E parallel stained sections. An average spectrum was taken for each BV, and all spectra were analyzed by chemometric methods using Opus 7.2 software (Bruker, Germany) and Unscrambler X 10.2 software (CAMO, Norway). Specifically, hierarchical cluster analysis (HCA) and principal component analysis (PCA) were the two chemometric methods that were applied to the data.

## Results

### Pixel binning

**Fig. 1:**
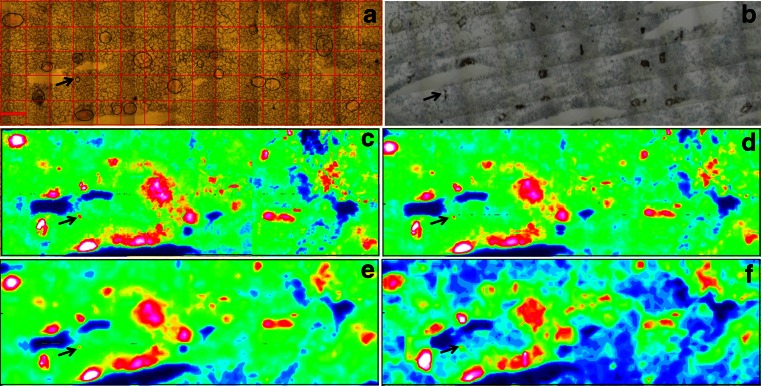
Mouse xenografted tumour section comparing different pixel binning modes. (**a**) Visible image of the tumour section deposited on ZnSe showing the FPA grid of about 2.3 mm^2^ area imaged with ×15 objective at 4 cm^−1^ and 8 scans co-added; BVs were *circled* for easy recognition and better comparison with the false colour maps. (**b**) Parallel section stained with CD31 to highlight all BVs. (**c**) Integration map binned at 2 × 2, with 81,920 spectra, effective pixel size of 5.3 μm, revealing clear BVs. (**d**) Integration map binned at 4 × 4, with 20,480 spectra, effective pixel size of 10.6 μm. (**e**) Integration map binned at 8 × 8, with 5120 spectra, effective pixel size of 21.3 μm. (**f**) PCA score 1 performed on binned 8 × 8 image as the number of spectra was reduced. The *black arrow* points to a small BV for which the structure is less clear with higher binning option. *Scale bar* (*red bar* in one FPA tile) in (**a**) is 171 μm

FTIR chemical images were achieved using an FPA multi-element detector, and large maps were a combination of several FPA tiles. A different pixel binning was performed on both sample types as described in Table 1. Figure 1 shows the mouse tumour section imaged in different binning modes, the effective pixel size increases, but the number of spectra and the acquisition time reduce when higher binning is performed. Therefore, the amount of data handling is reduced as well as the processing time for data analysis. From this figure, it is clear that there is no major difference between 4 × 4 binning and 2 × 2 binning, although the pixel projection on the sample is increased, which means that the spatial resolution is reduced. This may depend on the fact that the main IR features of interest were greater in size than the pixel projection size. This can be better seen with the 8 × 8 binning, where the resolution has decreased and the small BV of about 17 μm in diameter (indicated by black arrow) was giving less clear structure with respect to the original 2 × 2 binning acquisition. Still, a key advantage of binning is the smaller file size as the number of spectra is reduced. This can be helpful when chemometric analysis (e.g. HCA or PCA) is performed on large images where the high number of spectra limits the analysis capabilities of available/commercial IR software suites. For instance, using the Opus 7.2 software, the maximum number of spectra that can be handled in an IR map by PCA is about 8000 (i.e. two FPA tiles acquired without binning) while the limit for HCA analysis doubles the number of spectra (i.e. four FPA tiles). When binning reduced the number of spectra to roughly 5000 (Fig. 1e), PCA could be performed and the PC1 score plot (Fig. 1f) clearly reveals the BVs in the IR map.

It is also possible to perform binning post process; thus, it would be advisable to acquire FPA images without or with 2 × 2 binning to keep the highest spatial resolution in case of working on small features. Also, there is no practical advantage in acquisition time in mapping beyond 2 × 2 binning at the start as per Table 1.

### Human tumour sections

#### Small images

**Fig. 2:**
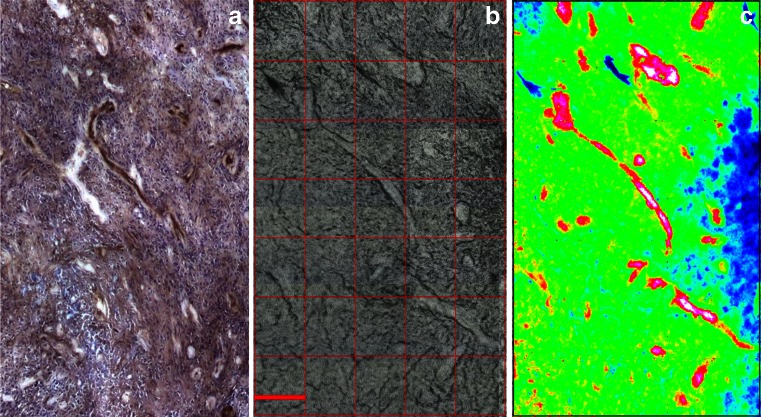
Human glioma section for small image S2 (GIV area). (**a**) IHC section stained with CD34. (**b**) Parallel section deposited on Si wafer that shows the visible image acquired with FPA with ×15 objective at 4 cm^−1^ and 32 scans without binning. (**c**) Protein-to-CH stretching ratio integration map of the FPA image. *Scale bar* (*red* in (**b**)) is 171 μm

**Fig. 3:**
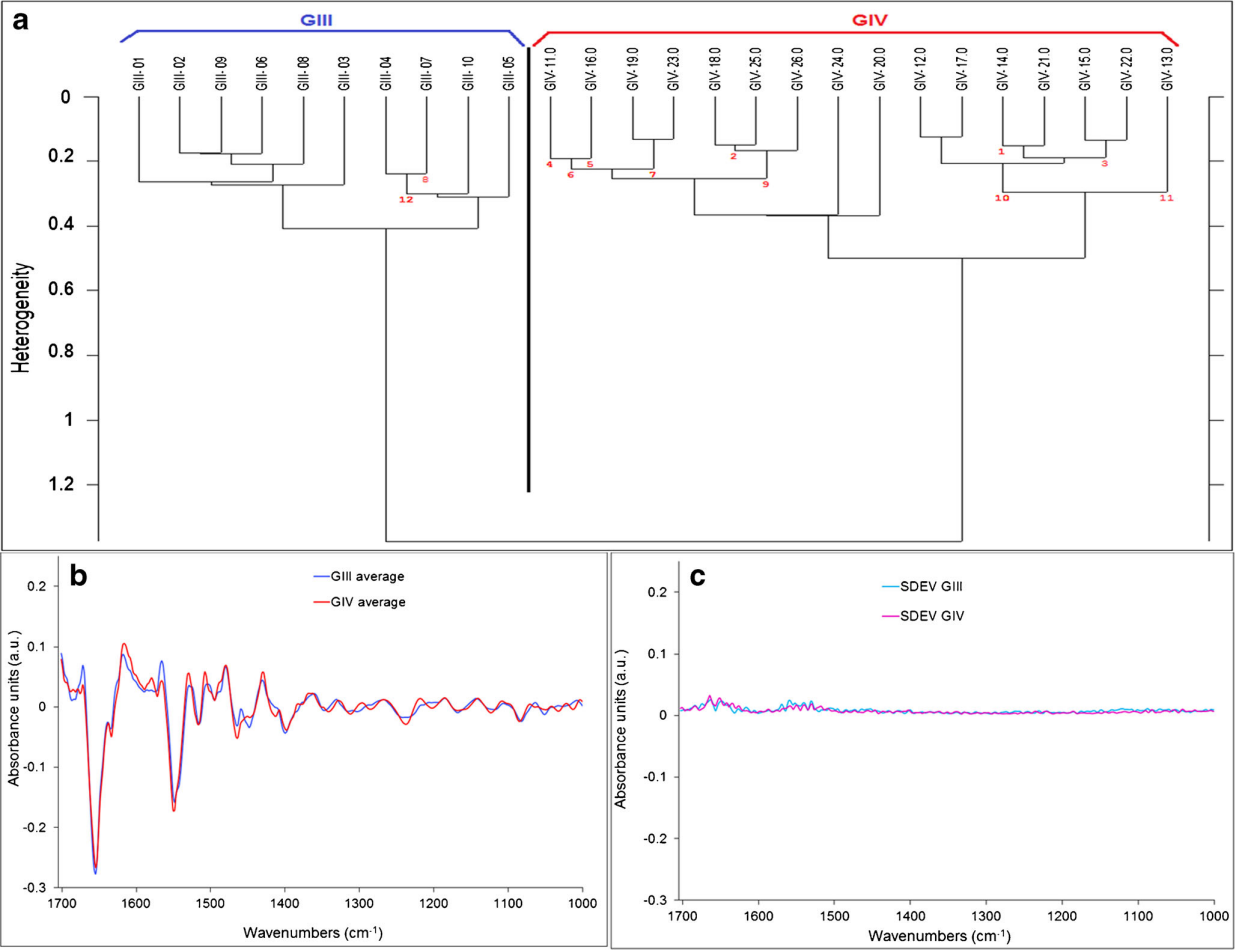
HCA dendrogram and average spectra graph for small human glioma images. (**a**) Dendrogram comparing BV spectra between the two small FPA images S1 GIII area (BVs 1–10) and S2 GIV area (BVs 11–26). Classification was based on the fingerprint region. (**b**) Average spectra of BVs from each group for the fingerprint region (1700–1000 cm^−1^). Spectra were second derivative, 13 points smoothed and vector normalized. (**c**) Standard deviation plots of the BV second derivative and vector-normalized spectra for each group for the fingerprint region. The same *scale* was respected in (**b**) and (**c**) graphs

These were acquired without binning to have the maximum resolution possible. Spectra from BVs were retrieved from the integration maps and averaged to have one average spectrum per BV. Image S1 was collected on an area identified by the pathologist as a GIII area. For this area, there were ten spectra representing BVs 1 to 10. Image S2 was collected on an area identified by the pathologist as a GIV sub-section far from the grade III sub-section. For this area, there were 16 spectra representing BVs 11 to 26. We were interested in comparing the characteristics of these different areas of gliomas referenced as *pure* grade III or IV using the IR spectral information of the BVs issued from the FPA detector image (Fig. 2, showing only image S2, image S1 is not shown for brevity). Spectra from these small images were then analyzed using the Opus 7.2 software by HCA using the second derivative 13-point Savitzky-Golay smoothing and vector normalization. The two main regions suggested to classify the spectra were the CH stretching (3000–2800 cm^−1^) and the fingerprint (1700–1000 cm^−1^) regions as these cover the main absorption bands in the full spectral range. The classification based on the fingerprint region alone gave two separate clusters where the BVs of each grade were grouped together without any misclassification (Fig. 3). The CH stretching region alone did not classify the two clusters perfectly; thus, the combination of the fingerprint and the CH stretching region did not improve the classification obtained by the fingerprint region alone. Therefore, we deduced that the main spectral region capable of separating the two clusters was the fingerprint region.

As can be seen in Fig. 3, the major differences between the second derivatives of the average spectra for each grade reside in the following sub-regions: 1700–1675, 1595–1585, 1555–1515, 1475–1450, 1290–1200 and 1050–1030 cm^−1^. Later, we subdivided the fingerprint region into three wave number intervals (1700–1500, 1500–1300 and 1300–1000 cm^−1^) to show precisely what the differences are between the two grades. These will be shown in the next paragraph to avoid repetition and also will be based on the larger number of spectra.

#### Bigger image

This IR map was acquired via 2 × 2 pixel binning in order not to compromise the spatial resolution for the sake of revealing smaller BV features. Spectra from BVs were retrieved from the protein-to-CH stretching ratio integration map and averaged to have one average spectrum per BV. This image contains two areas identified by the pathologists on the parallel section stained with H&E (see Electronic Supplementary Material (ESM) Fig. S1): one part as a GIII area (top part) and the other part as a GIV area (bottom part). It is worth mentioning that it is not always the case that pathologists can easily identify the grade or define the limits of the tumour. Both areas are separated in the H&E-stained section (see ESM Fig. S1, middle) due to cutting (the thinner section can be easily split during the cutting on the microtome or when deposited on the slide). There was no separation between the two areas in the thicker section deposited on Si wafer (see ESM Fig. S1, right) and imaged by IR; therefore, visually in this section, it makes it difficult to identify where the border between the two areas is.

##### HCA analysis

**Fig. 4:**
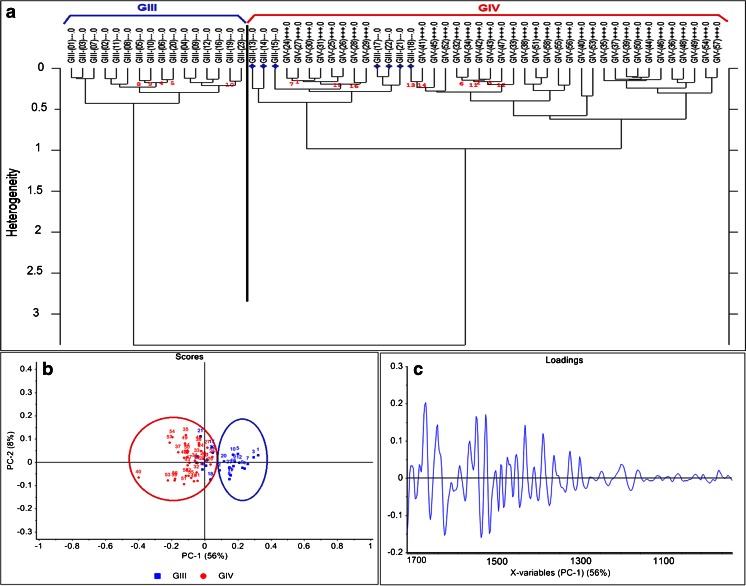
HCA dendrogram and PCA graphs for human glioma big image BV spectra. (**a**) HCA dendrogram comparing BV spectra between the two areas identified by the pathologists as GIII and GIV. GIV BV spectra are named with a *plus* sign to differentiate from GIII ones. Classification based on fingerprint region shows seven BVs (13–15, 17, 18, 21 and 22, marked by *blue stars*) from the GIII group classified in the GIV cluster. (**b**) PCA analysis confirms the misclassification of the same BVs obtained in the HCA. (**c**) Loading vector of PC1 which separates both groups based on the fingerprint region. Spectra were second derivative, 13 points smoothed and vector normalized

In the GIII area, there were 23 spectra identified for BVs numbered 1 to 23, and in the GIV area, there were 35 spectra for BVs numbered 24 to 58. These spectra were chosen after matching the two serial sections and drawing a borderline to separate the two grades based on the limit of the GIII area identified by the pathologist on the H&E section. Spectra were then analyzed using the Opus 7.2 software using the HCA method following the same procedure as for the small images in the previous data (i.e. second derivative 13-point Savitzky-Golay smoothing and vector normalization over the fingerprint region 1700–1000 cm^−1^). HCA gave two separate clusters where the BVs of each grade were grouped together with some misclassification (Fig. 4a). Seven BVs from the GIII area (named GIII since they originate from the area identified as GIII based on the pathology identification) were grouped with the GIV cluster. These were spectra 13, 14, 15, 17, 18, 21 and 22 (marked by blue stars in Fig. 4a).

##### PCA analysis

To confirm the classification was not due to the HCA method itself, PCA based on the fingerprint region was independently performed using the Unscrambler X 10.2 software and gave the same results as the HCA. PCA was performed also on the second derivative spectra, 13-points smoothed and vector normalized, using the non-linear iterative partial least squares (NIPALS) algorithm and leverage correction validation method. Figure 4b shows PCA based on PC1 versus PC2 scores where PC1 separates the two clusters, and the same seven BVs from GIII are grouped with the GIV cluster (blue numbers in the red circle). It is noteworthy that the influence Q-residual plot of PCA (referred as Hotelling *T*^2^ suited to spot samples which may be regarded as outliers for being too extreme in the model) grouped 97 % of the data in the well-described part. There were no *outliers* or *not well-described* samples.

##### Spectral markers

**Fig. 5:**
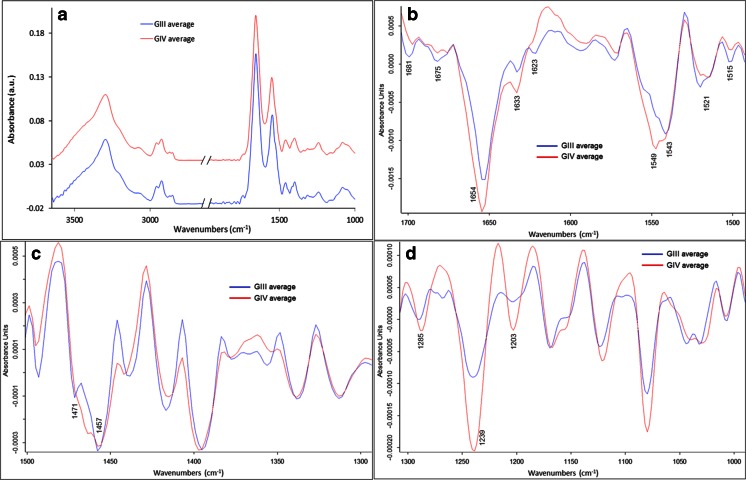
Average spectra graphs for human glioma big image of BVs of each group of GIII and GIV based on the classification in Fig. 4. (**a**) Absorbance spectra full range (spectra were offset for clarity). (**b**) Second derivative spectra in the region 1700–1500 cm^−1^. (**c**) Second derivative spectra in the region 1500–1300 cm^−1^. (**d**) Second derivative spectra in the region 1300–1000 cm^−1^. Spectra were 13 points smoothed and vector normalized. Peaks of interest are highlighted

The PC1 loading vector (Fig. 4c) could identify the differences between the two groups, but in order to highlight them better, second derivatives of the average spectra of each cluster were plotted together within the fingerprint range for each of the sub-regions previously mentioned (1700–1500, 1500–1300 and 1300–1000 cm^−1^) in Fig. 5.

In the 1700–1500 cm^−1^ sub-region corresponding to the protein region where amides I and II are the two major bands, few differences can be seen (Fig. 5b); the bands at 1681 cm^−1^ (more pronounced in GIII) and 1675 cm^−1^ (more pronounced in GIV, almost inexistent in GIII) differ between the two groups. These bands correspond to the β sheets and β turns, respectively, of the protein secondary structure [14, 19]. This can also be confirmed with the band at 1623 cm^−1^ (corresponding to β sheets) which is much more pronounced in GIII compared to GIV. The intensity of the bands at 1654 and 1633 cm^−1^ corresponding respectively to the α-helix and triple helix of amide I (C=O stretching) is higher in the GIV group with respect to the GIII group. The bands at 1549 and 1543 cm^−1^ as well as the bands at 1521 and 1515 cm^−1^ corresponding to the C–N stretching and N–H bending vibrations of the amide II band [20] change in the opposite direction between the two groups. These changes are induced by the conformational changes in the protein secondary structure in the BVs with the tumour progression.

In the 1500–1300 cm^−1^ range, where mostly lipids and proteins absorb, the major difference that can be seen (Fig. 5c) is around the two bands at 1471 and 1457 cm^−1^ corresponding respectively to the CH_2_ bending of the methylene chain in lipids [21] and the asymmetric CH_3_ bending mode of methyl groups in proteins [22]. The intensity of both bands decreased in the GIV group compared to the GIII group.

In the 1300–1000 cm^−1^ range, the major difference can be seen around the bands at 1285, 1239 and 1203 cm^−1^, which correspond to amide III of collagen [23], and these have higher intensity in the GIV group compared to the GIII group. This difference could be due to the structural alteration of collagen and its deformation with advanced tumour grade.

##### New boundary between the two grades

**Fig. 6:**
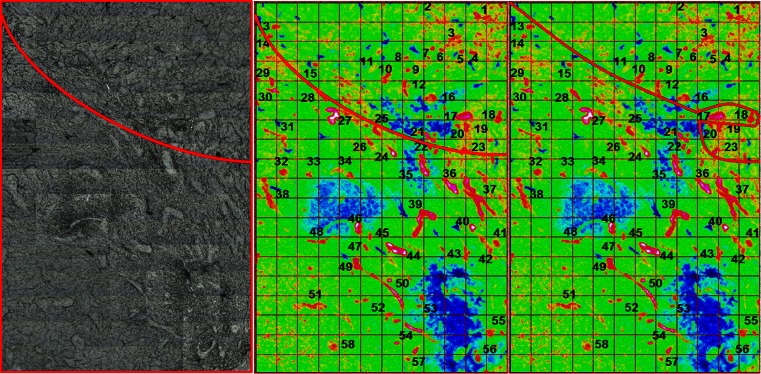
Human glioma big image integration map. *Left* visible image acquired with the FPA where there are two areas [GIII (*top*) and GIV (*bottom*) separated by the *red line*]. *Middle* protein-to-CH stretching ratio integration map of the FPA image showing the BVs with their assigned *numbers* and a *red line* splitting between the two grades as identified by the pathologists. *Right* the same integration map but with a new *red line* separating the two areas based on IR spectra classification by HCA and PCA analysis

Following the classification of the IR spectra retrieved from the bigger image of the human tissue section with the two areas of GIII and GIV being in contact, a new boundary between the two grade areas could be drawn. This is shown in Fig. 6. First, the left panel shows the visible image of the tissue section part which was acquired by the FPA with the red line defining the borderline between the two areas identified by the pathology observation. The second mid panel shows the protein-to-CH stretching-integrated band ratio as a false colour IR intensity map with the same red line drawn that separates between the BVs of the GIII and GIV areas. In the far right panel, the same area is shown with a new red line drawn separating the two areas based on the HCA and the PCA classification of the BV IR spectra. This was done by taking into consideration the seven BVs originally labelled as GIII from the pathologist’s observation but then classified as GIV after IR analysis and chemometrics with both methods. This mismatch could be due to two reasons: (i) glioblastomas are known to be very invasive and highly heterogeneous tumours; hence, a mix of grades, especially for the advanced stages, is possible; (ii) progression from GIII to GIV as described before is consistent with mixed BV spectra at the limit between the two grades. In both cases, the molecular information of these BVs that could be revealed by FTIR imaging helped to identify the borderline between the two areas. It was not straightforward to do this when comparing with the H&E-stained section (used for pathology) where the two areas were separated and not completely in contact.

## Discussion

The accurate classification of human gliomas has many implications for the care of patients in estimating prognosis and guiding therapy [24]. Assigning a precise grade is sometimes not possible. Diffuse glioma grading is based on the World Health Organization (WHO) criteria for astrocytomas and oligodendrogliomas [25]. Pathological definitions of the grades, e.g. atypia, mitoses, microvascular proliferation and necrosis, could help the pathologist to diagnose the tumour as being at least WHO grade III. Clinical features are similar for grade III glioma and diffuse glioma WHO grade II. Sometimes with a prior history of a diffuse grade II astrocytoma, there are increasing neurological deficits. Some patients have a shorter course without previous history of a glioma. For glioblastoma WHO grade IV, the clinical history is usually short (less than 3 months in more than 50 %). Surgery, radiotherapy and chemotherapy are the treatments available for the purpose, and there are treatment specificities between glioma grades and types; if the tumour is reachable, surgery is recommended as a first step to remove as much as possible from the tumour knowing that complete resection is often not feasible. For WHO grade III glioma, it depends if it is an anaplastic grade III oligodendroglioma or an oligoastrocytoma (radiotherapy is preferred for little tumours, whereas radio-chemotherapy is preferred for larger ones) or astrocytoma (radiotherapy is the standard) [26, 27]. For glioblastoma WHO grade IV, the Stupp design is the reference which associates radiotherapy and temozolomide along with a specific plan [28].

Pathologist’s observation is currently accredited as the accurate tool to identify alterations in the structure of diseased tissues, and glioma tumour classification depends on the experience of the observer and is subject to optimal sample contrast. In spite of using stained sections, sometimes it is difficult for the pathologist to strictly define the grade of a tumour and this can affect the patient follow-up treatment. Thus, a complementary objective technique is needed in this sense. Since the progression of the glioma tumours largely depends on the angiogenesis and the microvascular proliferation, the key idea is to study the BVs of these tumours by vibrational spectroscopy to see if this can help to identify different issues. The hypothesis was partially tested in previous published work. The continuation is in progress to study different grades and increase the grading factor library of these tumours based on their vascularization. Apart from previous work published by these authors, no other work has been reported in this field. However, there are parallel works done by few groups to study the bulk brain tumour tissue. Gajjar et al. reported that in studying different types/grades of brain tumours by IR and Raman spectroscopy, they were able to segregate various grades of glioma more readily compared to current staining methods [21]. Other studies [29, 30] also showed the potential application of IR spectroscopy in diagnosing and grading gliomas based on the tumour tissue.

In this work, FTIR-FPA imaging was implemented to specifically study the tumour vascularization in order to prove its potential to detect biochemical changes in the tumour tissue which can be helpful for grading the tumour. Current technology of FPA detector permits to quickly acquire IR maps over large areas of tissue sections already in its standard version with 64 × 64 pixel matrix. This is useful for rapid screening of large tissue components and/or localization of areas of biochemical interest. Good spectral quality is then provided at single pixel size in which its projected size varies from 2.6 μm (×15 objective) to multiple of 2 when binning is performed. Here, two types of samples were studied: (i) the mouse tumour data served only as a test to determine which binning option is the optimal. This was done to assess FTIR imaging via an FPA detector and globar source in terms of performances when analyzing large tumour tissue sections using different binning modes without compromising the IR image information content. We realized that binning beyond 2 × 2 was not beneficial for our specific samples, given the size of the features of interest (as a rule of thumb, these should not be smaller than the pixel projection size). If working on large features, binning of 8 × 8 could be performed to reduce the file size and perform further data analysis rapidly. (ii) The second sample was a human glioma tissue. The aim was to check the reliability of IR imaging via FPA screening when implementing it to analyse large areas of glioma tissue section of two different grades, i.e. astrocytoma GIII and glioblastoma GIV. The use of FPA detector for IR mapping combined with chemometric analysis allowed us to highlight BV spectroscopic markers not detectable by conventional H&E or IHC staining. These markers helped to identify the borderline between two close areas of different grades (III and IV) in glioma tissue sections. The biomolecular markers from the BVs, revealed in the IR fingerprint spectral region (1700–1000 cm^−1^), could be used as discriminating factors for differentiating the areas of different glioma grades.

Regarding the data of the human glioma tumour, it is quite rare to find a sample as the one studied in this work which shows different grades in different areas (two separated regions and two adjacent regions). Despite of having only one sample that can limit the impact of the study, we believe that this is a valid proof of principle for using IR imaging in glioma grading. In particular, the spectral analysis based on a relative comparison of different glioma grades on the same specimen from the same patient overcomes the intrinsic complication given by the bio-variability that the use of samples from different patients with only one/two specific grades could introduce, especially for this kind of tumours known to be very heterogeneous as previously mentioned. Although the human brain samples are some of the most difficult to get hold of, more samples with concomitant different grades from more patients will be studied as soon as available. For this same patient, IR imaging via an FPA detector was able to separate the graded areas as GIII and IV both when spatially well separated and adjacent. The major outcome of the work is the proof of concept as the potential advantage of IR microanalysis over the pathological identification to establish the borderline between the two graded areas based on the molecular spectra, information not available by visual microscopy where only the morphological aspect is evaluated.

## Conclusion

The results of this project are a promising step forward for implementing FTIR-FPA imaging as a reliable complementary label-free technique to grade gliomas. The revealed spectral biomolecular markers from the glioma vascularization could be used as discriminating factors for differentiating the areas of different glioma grades. This study represents the proof of principle that a complementary spectroscopy technique like FTIR—not based only on visual observation and experienced observer—is a helpful tool for this specific biomedical research on tumours. Although the data are limited, this work clearly shows the potential advantage of FTIR microanalysis over the pathological identification in defining the fine borderline between two differently graded tumoural tissue areas. More samples with concomitant different grades and from more patient cases will be studied in the future.

## Electronic supplementary material

ESM 1 (PDF 2168 kb)

## References

[1] Kleihues P, Louis DN, Scheithauer BW, Rorke LB, Reifenberger G, Burger PC, Cavenee WK (2002). The WHO classification of tumors of the nervous system. J Neuropathol Exp Neurol.

[2] Nakamura M, Shimada K, Nakase H, Konishi N (2009). Clinicopathological diagnosis of gliomas by genotype analysis. Brain Nerve.

[3] Figarella-Branger D, Bouvier C (2005). Histological classification of human gliomas: state of art and controversies. Bull Cancer.

[4] Parolaro D, Massi P (2008). Cannabinoids as potential new therapy for the treatment of gliomas. Expert Rev Neurother.

[5] Puduvalli VK, Sawaya R (2000). Antiangiogenesis—therapeutic strategies and clinical implications for brain tumors. J Neuro-Oncol.

[6] Plate KH, Risau W (1995). Angiogenesis in malignant gliomas. Glia.

[7] Walker C, Baborie A, Crooks D, Wilkins S, Jenkinson MD (2011). Biology, genetics and imaging of glial cell tumours. Br J Radiol.

[8] Van den Bent MJ (2010). Interobserver variation of the histopathological diagnosis in clinical trials on glioma: a clinician’s perspective. Acta Neuropathol.

[9] Bhargava R (2007). Towards a practical Fourier transform infrared chemical imaging protocol for cancer histopathology. Anal Bioanal Chem.

[10] Baker MJ, Trevisan J, Bassan P, Bhargava R, Butler HJ, Dorling KM, Fielden PR, Fogarty SW, Fullwood NJ, Heys KA, Hughes C, Lasch P, Martin-Hirsch PL, Obinaju B, Sockalingum GD, Sule-Suso J, Strong RJ, Walsh MJ, Wood BR, Gardner P, Martin FL (2014). Using Fourier transform IR spectroscopy to analyze biological materials. Nat Protoc.

[11] Beljebbar A, Dukic S, Amharref N, Manfait M (2010). Screening of biochemical/histological changes associated to C6 glioma tumor development by FTIR/PCA imaging. Analyst.

[12] Ali K, Lu Y, Das U, Sharma RK, Wiebe S, Meguro K, Sadanand V, Fourney DR, Vitali A, Kelly M, May T, Gomez J, Pellerin E (2010). Biomolecular diagnosis of human glioblastoma multiforme using Synchrotron mid-infrared spectromicroscopy. Int J Mol Med.

[13] Wehbe K, Pineau R, Eimer S, Vital A, Loiseau H, Deleris G (2010). Differentiation between normal and tumor vasculature of animal and human glioma by FTIR imaging. Analyst.

[14] Wehbe K, Pinneau R, Moenner M, Deleris G, Petibois C (2008). FT-IR spectral imaging of blood vessels reveals protein secondary structure deviations induced by tumor growth. Anal Bioanal Chem.

[15] Wehbe K, Travo A, Eimer S, Cinque G, Barron E, Deleris G, Forfar I (2013). Investigation of blood vessels in glioblastoma at a micrometric scale: a comparative study by synchrotron and conventional micro-FTIR. Anal Methods.

[16] Dorling KM, Baker MJ (2013). Rapid FTIR chemical imaging: highlighting FPA detectors. Trends Biotechnol.

[17] Bassan P, Sachdeva A, Shanks JH, Brown MD, Clarke NW, Gardner P (2013). Whole organ cross-section chemical imaging using label-free mega-mosaic FTIR microscopy. Analyst.

[18] Bassan P, Sachdeva A, Shanks JH, Brown MD, Clarke NW, Gardner P (2014). Automated high-throughput assessment of prostate biopsy tissue using infrared spectroscopic chemical imaging. Proc SPIE.

[19] Bonnier F, Rubin S, Debelle L, Venteo L, Pluot M, Baehrel B, Manfait M, Sockalingum GD (2008). FTIR protein secondary structure analysis of human ascending aortic tissues. J Biophotonics.

[20] Kong J, Yu S (2007). Fourier transform infrared spectroscopic analysis of protein secondary structures. Acta Biochim Biophys Sin (Shanghai).

[21] Gajjar K, Heppenstall LD, Pang W, Ashton KM, Trevisan J, Patel II, Llabjani V, Stringfellow HF, Martin-Hirsch PL, Dawson T, Martin FL (2013). Diagnostic segregation of human brain tumours using Fourier-transform infrared and/or Raman spectroscopy coupled with discriminant analysis. Anal Methods.

[22] Chen YJ, Cheng YD, Liu HY, Lin PY, Wang CS (2006). Observation of biochemical imaging changes in human pancreatic cancer tissue using Fourier-transform infrared microspectroscopy. Chang Gung Med J.

[23] Belbachir K, Noreen R, Gouspillou G, Petibois C (2009). Collagen types analysis and differentiation by FTIR spectroscopy. Anal Bioanal Chem.

[24] Louis DN, Perry A, Burger P, Ellison DW, Reifenberger G, von Deimling A, Aldape K, Brat D, Collins VP, Eberhart C, Figarella-Branger D, Fuller GN, Giangaspero F, Giannini C, Hawkins C, Kleihues P, Korshunov A, Kros JM, Beatriz Lopes M, Ng HK, Ohgaki H, Paulus W, Pietsch T, Rosenblum M, Rushing E, Soylemezoglu F, Wiestler O, Wesseling P (2014). International Society Of Neuropathology—Haarlem consensus guidelines for nervous system tumor classification and grading. Brain Pathol (Zurich, Switzerland).

[25] Louis DN, Ohgaki H, Wiestler OD, Cavenee WK, Burger PC, Jouvet A, Scheithauer BW, Kleihues P (2007). The 2007 WHO classification of tumours of the central nervous system. Acta Neuropathol.

[26] Cairncross G, Wang M, Shaw E, Jenkins R, Brachman D, Buckner J, Fink K, Souhami L, Laperriere N, Curran W, Mehta M (2013). Phase III trial of chemoradiotherapy for anaplastic oligodendroglioma: long-term results of RTOG 9402. J Clin Oncol.

[27] Van den Bent MJ, Brandes AA, Taphoorn MJ, Kros JM, Kouwenhoven MC, Delattre JY, Bernsen HJ, Frenay M, Tijssen CC, Grisold W, Sipos L, Enting RH, French PJ, Dinjens WN, Vecht CJ, Allgeier A, Lacombe D, Gorlia T, Hoang-Xuan K (2013). Adjuvant procarbazine, lomustine, and vincristine chemotherapy in newly diagnosed anaplastic oligodendroglioma: long-term follow-up of EORTC brain tumor group study 26951. J Clin Oncol.

[28] Stupp R, Mason WP, van den Bent MJ, Weller M, Fisher B, Taphoorn MJ, Belanger K, Brandes AA, Marosi C, Bogdahn U, Curschmann J, Janzer RC, Ludwin SK, Gorlia T, Allgeier A, Lacombe D, Cairncross JG, Eisenhauer E, Mirimanoff RO (2005). Radiotherapy plus concomitant and adjuvant temozolomide for glioblastoma. N Engl J Med.

[29] Steiner G, Shaw A, Choo-Smith LP, Abuid MH, Schackert G, Sobottka S, Steller W, Salzer R, Mantsch HH (2003). Distinguishing and grading human gliomas by IR spectroscopy. Biopolymers.

[30] Krafft C, Sobottka SB, Geiger KD, Schackert G, Salzer R (2007). Classification of malignant gliomas by infrared spectroscopic imaging and linear discriminant analysis. Anal Bioanal Chem.

