# Supplementary material for: Discrimination between two different grades of human glioma based on blood vessel infrared spectral imaging
Source: Anal Bioanal Chem. 2015 Jul 14;407(24):7295–305. doi: 10.1007/s00216-015-8891-z (PMC4569654; doi:10.1007/s00216-015-8891-z)
Supplement: Supplementary file 1 — (PDF 2168 kb) [file 216_2015_8891_MOESM1_ESM.pdf]

Analytical and Bioanalytical Chemistry

Electronic Supplementary Material

Discrimination between two different grades of human glioma based on blood vessel Infrared spectral imaging

Katia Wehbe, Isabelle Forfar, Sandrine Eimer, Gianfelice Cinque

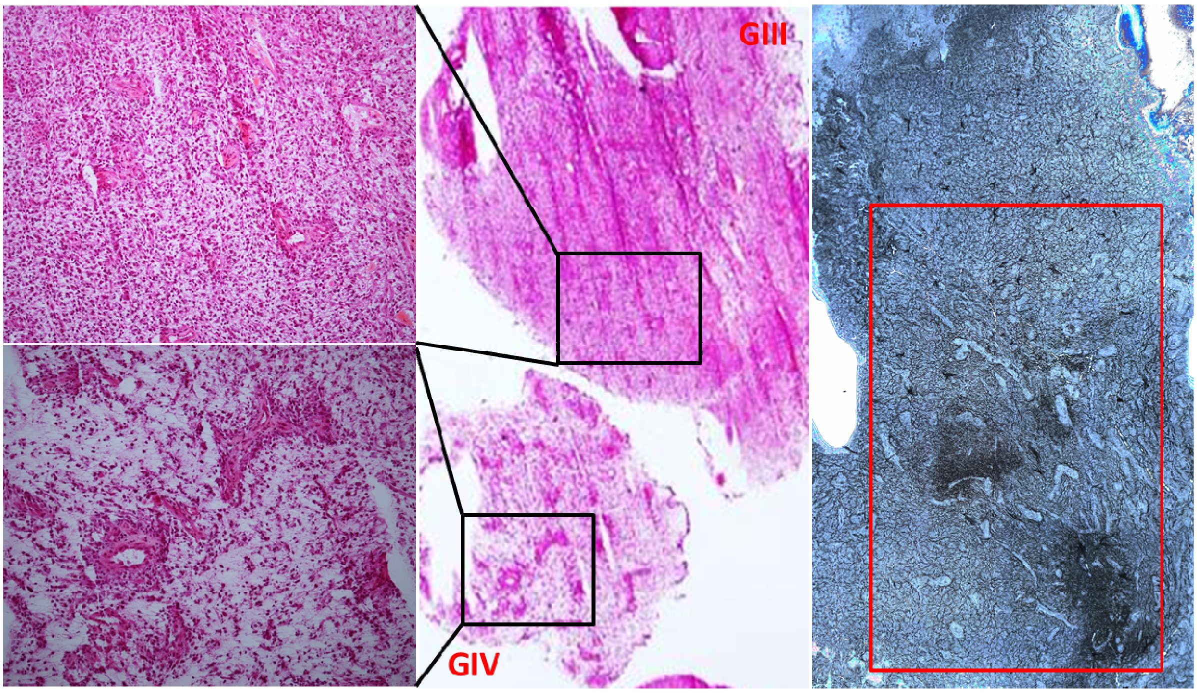

Fig. S1 Human Glioma section for big image. Left and middle: H&E stained section where the pathologists identified GIII and GIV areas (black boxes are magnified to the left to show the BVs and the cellular density). Right: Visible image of the parallel section deposited on Si wafer on which the area of the big image was chosen to be acquired with the FPA (shown in the red square)
